# The Role of the Extracellular Matrix Components in Cutaneous Wound Healing

**DOI:** 10.1155/2014/747584

**Published:** 2014-03-17

**Authors:** Pawel Olczyk, Łukasz Mencner, Katarzyna Komosinska-Vassev

**Affiliations:** ^1^Department of Community Pharmacy, Medical University of Silesia, ul. Kasztanowa 3, 41-200 Sosnowiec, Poland; ^2^Department of Clinical Chemistry and Laboratory Diagnostics, Medical University of Silesia, ul. Jednosci 8, 41-200 Sosnowiec, Poland

## Abstract

Wound healing is the physiologic response to tissue trauma proceeding as a complex pathway of biochemical reactions and cellular events, secreted growth factors, and cytokines. Extracellular matrix constituents are essential components of the wound repair phenomenon. Firstly, they create a provisional matrix, providing a structural integrity of matrix during each stage of healing process. Secondly, matrix molecules regulate cellular functions, mediate the cell-cell and cell-matrix interactions, and serve as a reservoir and modulator of cytokines and growth factors' action. Currently known mechanisms, by which extracellular matrix components modulate each stage of the process of soft tissue remodeling after injury, have been discussed.

## 1. Introduction

Wound healing is a complex, biological process which concerns replacing damaged tissue by a living one [1–3]. The restoration of tissue integrity is the result of the interaction of platelets, cells, such as neutrophils, monocytes/macrophages, fibroblasts, endothelial cells, and keratinocytes as well as extracellular matrix (ECM) components, such as fibronectin, glycosaminoglycans, proteoglycans, thrombospondins, tenascin, vitronectin, or collagens [4, 5]. The mentioned cell interaction with ECM components is subject to a regulation of biochemical mediators, numerous cytokines, and growth factors, such as arachidonic acid derivatives (prostaglandins and leukotrienes), interleukins, interferons, TNF-*α*, PDGF, FGF, TGF, or EGF [6]. First of the mentioned compounds participates in creating the inflammatory response, while the others, that is, growth factors, take part in controlling proliferation, differentiation, and metabolism of cells involved in the healing process. The latter mediators assist in regulating inflammatory processes and play a chemotactic role for neutrophils, monocytes/macrophages, fibroblasts, and epithelial cells (keratinocytes) stimulating the angiogenesis and formation of ECM [7, 8].

The delicate balance between the above mentioned processes—proliferation and differentiation—is regulated by stem cells capable of enhancing the repair via secretion of paracrine factors [9, 10]. Endothelial progenitor cells, derived from hematopoietic stem cell lineage, play a key role in the neovascularization [3]. It was also observed that conditioned media obtained from mesenchymal stem cells promote wound healing through activation of host cells. Furthermore, topical application of mesenchymal stem cells enhances chronic wound healing and implementation of recombinant cytokines secreted by stem cells could be beneficial for recalcitrant wounds [10]. Moreover, bone-marrow mesenchymal stem cells may differentiate into fibroblasts and keratinocytes—cells responsible for synthesis of ECM constituents [3, 11–13].

ECM components play a significant role in each stage of the healing process. It concerns, on one hand, the structural biomechanical aspect of the process in question because the ECM components create “scaffolding” (a temporary matrix, granulation tissue, and scar), which is indispensable in the repairing process, providing in this way a structural integrity of the matrix during each stage of the healing process [5, 12–14]. On the other hand, however, the role of ECM components is connected with the action aspect of the healing processes since the mentioned compounds also fulfill a function of signal transduction in this dynamic, interactive sequence of biological reactions [15–20]. The latter functions are connected with stimulating the adhesion and migration of cells during the healing process as well as with mediating the interactions among cells, between cells and the matrix, or between ECM proteins [12, 13, 17, 21]. ECM components serve also as a reservoir and a modulator of cytokines and growth factors' action, thus regulating wound repair activity [5, 15, 22–24].

Dermatan sulfate enhances endothelial leukocyte adhesion by the stimulation of ICAM-1 or fibroblast growth factor-2 as well as it participates in the interaction with hepatocyte growth factor/scatter factor heparin cofactor II, platelet factor 4, fibronectin, or protein C inhibitor [25–28]. Chondroitin sulfate is able to induce FGF-2-mediated cell proliferation, control cell adhesion, and stimulate cell spreading and migration by activating focal adhesion of growth factor [28, 29]. Heparan sulfate/heparin participates in regulation of angiogenesis, cell growth, migration, and differentiation [30–32]. Hyaluronic acid determines tissue hydration, functions as a signaling molecule, interacts with cell surface receptors, and stimulates cell proliferation, migration, differentiation, and gene expression [28, 33].

The wound healing of skin is different from the process of damaged bone repair [34, 35]. Skin wounds heal by first intention or granulation [6]. So, a surgically sewn cut, which is not accompanied by tissue loss, leads to healing by first intention. Greater wounds, including postburn injuries, in which tissue loss and infection of the damage take place, heal by granulation. In the mentioned case, the final effect of healing is a scar.

Wound healing proceeds through four, but overlapping, phases, such as hemostasis, inflammation, proliferation (also known as replication and synthesis stage), and remodeling [3, 36]. 4 stages were created because of practical reasons, while the division itself has an arbitrary character because subsequent stages overlap as before one stage finishes, the next one starts [37, 38].

**Figure 1:**
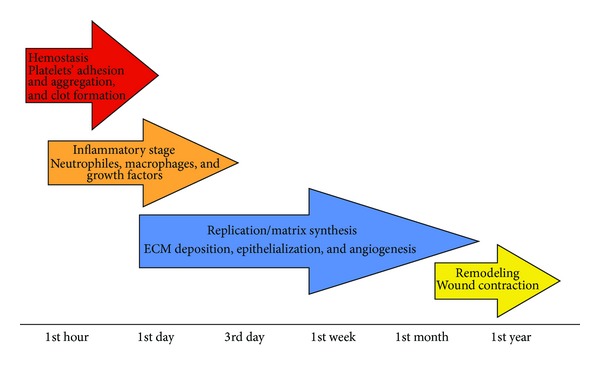
Healing stages.

Healing skin wounds proceeds in accordance with the mentioned below stages (Figure 1).

## 2. Healing Stages

### 2.1. Hemostasis

The first stage of wound healing starts immediately after an injury appears [36]. It begins with narrowing the damaged vessels, which is caused by the activity of vasoconstriction factors, such as serotonin, thromboxane A_2,_ or adrenaline being, on the other hand, connected with adhesion, aggregation, and platelets' activation in the damaged place [37].

The platelets are early modulators of the healing process [15]. They undergo adhesion, aggregation, and activation as a result of their contact with collagen of the damaged vessels, which leads to ADP and adhesion glycoprotein release from them which in turn supports further platelet aggregation [37]. The key glycoproteins, which are released from *α* granules of platelets, are fibrinogen, fibronectin, vitronectin, thrombospondin, and von Willebrand's factor [39, 40]. The surface of the activated platelets simultaneously becomes the place of prothrombin activation, which leads to creation of active thrombin—the key factor of the coagulation process catalyzing the transformation of fibrinogen into fibrin and as a result of that it forms a blood clot [35, 39, 40].

The blood clot protects the structural integrity of vessels and provides a provisional “scaffolding” which enables formation of a temporary matrix in the wound bed. Besides fibrin molecules, the main component of this temporary, hyaluronan-rich matrix is also plasma fibronectin, which is accumulated in the wound during the first 24 hours after the injury [41]. The polymerized fibronectin shows highly adhesive properties entering the interaction with numerous cells by integrin receptors and stimulates the migration and adhesion of fibroblasts, keratinocytes, and endothelial cells. Being one of the ligands for platelet integrin, it supports further adhesion and aggregation of these morphotic elements [42]. The aggregated platelets, “trapped” in the provisional matrix, release, from *α* granules, numerous growth factors, such as PDGF, TGF-*α*, TGF-*β*, bFGF, IGF-1, and VEGF [3, 35, 36, 43]. These mediators influence neutrophils, monocytes/macrophages, smooth muscle cells, endothelial cells, and fibroblasts [3]. So, neutrophils and monocytes are recruited into the wound environment by PDGF and TGF-*β*, which is to initiate the inflammatory response [15, 44]. Additional, chemotactic stimuli intensifying the recruitment of neutrophils are products of C5a complement degradation as well as the products of bacteria decomposition. Endothelial cells are activated by TGF-*α*, Bfgf, and VEGF in order to initiate angiogenesis. Fibroblasts, in turn, are activated and recruited by PDGF and IGF-1 in order to initiate the migration of these cells into the wound environment and their proliferation as well as biosynthesis of glycosaminoglycans and collagen [36, 44].

Summing up, the healing process is initiated by the hemostasis stage, which is connected with forming a temporary matrix, secreting cytokines and other growth factors, and interaction of the latter ones with ECM, which initiates the repairing process, preparing the wound bed to the next stage of the healing process—the inflammatory stage [36, 37].

### 2.2. Inflammatory Stage

Inflammatory phase of the healing process develops during 24 hours from the moment when an injury occurred and lasts for up to 48 hours on average [34]. This phase is accompanied by characteristic inflammatory symptoms, such as redness, body heat, swelling, and pain around the wounded place [34]. The early inflammatory phase of the wound healing cascade is characterized by subsiding of the initial vessel contraction followed by widening their lumen with accompanying increased vascular permeability of walls, which promotes “leaking” of plasma to the wounded tissue area [2, 6, 45]. The changes are supported by histamine, kinins, and prostaglandins and, moreover, leukotrienes, proteases, acid hydrolases, nitrogen oxide, and reactive oxygen species [3, 37]. The latter one is a major stimulus of VEGF synthesis and provides a substantial role in the immune defence in the wound [46]. In our previous works, different types of free radicals were found in the burn wounds samples by application of an innovatory numerical procedure of spectroscopic skin analysis such as continuous microwave saturation of multicomponent electron paramagnetic resonance spectra. The effect of microwave power on the asymmetry parameters of the spectra indicated the complex character of free radical system in the tested samples and allowed to obtain the major information about multicomponent structure of free radical system in burn wounds [47, 48].

The key cells of the inflammatory phase are neutrophils and monocytes/macrophages [2, 6, 45]. These cells in addition to keeping the wound aseptic by active phagocytosis and debridement, they simultaneously release a large number of active mediators (cytokines and growth factors), the action of which is crucial to initiate the next phase of the healing process [3, 37, 38, 49, 50].

Neutrophils are the first inflammatory cells which appear in the wound area. The recruitment of these cells takes place a few minutes after the injury [35, 39, 51]. Under the influence of chemotactic factors, such as thrombin, products of fibrin decomposition, bacteria, complement (C5a) components, histamine, PGE_2_, leukotrienes, TGF-*β*, and PDGF, neutrophils are “attracted” to the place of damage [3, 39, 50]. These cells create the first line of defence against infections phagocytising and killing the bacteria by generating reactive oxygen and nitrogen species and digesting, by released proteases (elastase, collagenase, and cathepsin G) the damaged, during the injury, connective tissue components [52–54].

The cells in question intensify the inflammatory reaction by releasing proinflammatory cytokines—IL-1 and TNF-*α* [37]. After two- or three-day presence in the wound area, the neutrophils are depleted in the process of apoptosis and are replaced by monocytes [36, 37].

Monocytes migrate from capillary to ECM where, under the influence of inflammatory mediators, such as TGF- *β* and products of fibrin and fibronectin degradation coming from the “temporary” wound matrix, they undergo a transformation into macrophages [3]. The chemotactic and mitogenic factor for monocytes/macrophages is, furthermore, thrombin [39]. The influx of the inflammatory cells in question to the wound area begins on the first day after the tissue injury, while, after 48 hours, they become the dominating inflammatory cells in the wound bed [34, 39].

Macrophages are cells of a great importance for the process of healing [3, 49, 55, 56]. Similar to neutrophils, macrophages play a double role in the healing process [3, 49, 57, 58]. On one hand, they participate in phagocytosis and process of killing bacteria or removing debris, by secreting matrix metalloproteinases, for example, collagenase, or elastase; on the other hand, however, they are the main source of cytokines and growth factors stimulating the proliferation of fibroblasts and collagen biosynthesis [3, 36, 57–59]. Releasing the plasminogen activator, they cause the removal of fibrin cloth. Moreover, they are the source of TGF-*β* also secreting PDGF, TGF-*α*, bFGF, HB-EGF, IL-1, IL-6, and TGF-*α* [15, 50]. The mentioned mediators do not only control the inflammatory process but also modulate the epithelialization, collagen accumulation, and angiogenesis [35, 37, 39, 60].

In the late inflammatory phase, lymphocytes also infiltrate the wound environment influencing fibroblast proliferation and collagen biosynthesis [3].

Summing up, the inflammatory phase, which is initiated by neutrophils and developed under the influence of macrophages, is connected with cleansing the bacteria and debris remains from the wound area as well as with releasing from the mentioned inflammatory cells soluble mediators, such as proinflammatory cytokines (IL-1, IL-6, IL-8, and TNF-*α*) and growth factors (PDGF, TGF-*α*, TGF-*β*, IGF-1, and FGF) responsible for recruitment and activation of fibroblasts and epithelial cells creating in this way conditions for initiating the next phase of the healing process [49, 55, 61–63].

The absence of neutrophils as well as the reduced amount of macrophages in the wound environment indicates that the inflammatory phase comes to an end and the proliferation phase starts [15].

### 2.3. Proliferation Phase

After hemostasis and inflammatory phases, which have lasted from 2 to 3 days, the process of rebuilding the damaged tissue is intensified [3]. During this time, the number of cells in the wound bed increases, which is connected with migration and proliferation of fibroblasts and endothelial cells as well as keratinocytes. The first of them—fibroblasts—secretes IGF-1, bFGF, TGF-*β*, PDGF, and EGF. Endothelial cells synthesize VEGF, bFGF, and PDGF, while keratinocytes synthesize TGF-*α*, TGF-*β*, and KDAF (an autocrine factor that derives from keratinocytes). The mentioned mediators stimulate and modulate (a) ECM biosynthesis, (b) epithelialization, and (c) angiogenesis [6].

*(a) ECM Biosynthesis.* A temporary matrix, formed mainly from fibrin and fibronectin network is replaced by collagen matrix, enriched in proteoglycans, glycosaminoglycans, and noncollagenous glycoproteins, which further lead to restoring the structure and function of the proper tissue [4].

The key cells of the discussed phase are fibroblasts. They are formed mainly from nondifferentiated mesenchymal cells, residing in the dermis, which, under the influence of cytokines and growth factors, released from blood platelets, neutrophils, and macrophages, undergoes a transformation into fibroblasts [3, 64, 65]. These cells migrate to the place of damage during 48–72 hours from the moment when the injury appears [34]. The cells in question are “attracted” to the wound area according to the chemotactic PDGF, EGF, IGF-1, and TGF-*β* gradient where the proliferation of these cells takes place (stimulated by growth factors) and, after that the synthesis of ECM components and formation of “granulation” tissue starts [24, 39, 66, 67]. The term “granulation” comes from a specific, granulated look of the newly formed connective tissue framework which is “intertwined” by many capillaries [68]. This tissue appears around the fourth day after the injury [69].

The granulation tissue is created by collagen (mainly types I and III), elastin, proteoglycans, glycosaminoglycans, and noncollagenous proteins synthesized mainly by fibroblasts whose activity is regulated by PDGF and TGF-*β* [70–72]. The first of the mentioned growth factors, originating mainly from blood platelets and macrophages, stimulates also the expression of collagenase, while the second one, which is also secreted by blood platelets and macrophages, regulates the accumulation of ECM components [15]. The matrix of the early granulation tissue (up to the third day after the injury) contains great amount of hyaluronic acid and fibronectin. The hyaluronic acid molecules, which are characterized by an ability to swelling, create a woven structure which enables the coming cells to penetrate the wound area [70]. Fibronectin, however, creates “scaffolding” facilitating the fibrogenesis of collagen [34]. Starting with the third day after the injury, the concentration of hyaluronic acid within the wound area quickly decreases, while collagen takes the place of this glycosaminoglycan. The collagen content in the granulation tissue increases up to the third week, from the moment when the wound appeared, which is accompanied by a gradual decrease of the fibroblast amount up to the moment when they disappear in the process of apoptosis [73]. In the dermis, the dominating types of collagens are types I and III which occur in a proportion of 4 : 1. During the initial phases of healing, however, collagen type III predominates. This protein “toughens” the newly created tissue giving it the feature of tensile strength [39]. The matrix of granulation tissue is also enriched in heparan sulfate proteoglycans, which appear in the wound area after a few hours from the injury [74], as well as chondroitin/dermatan proteoglycans, which appear in the wound area significantly later in the second week of the healing process [28, 70]. The granulation tissue, temporarily substituting the dermis, ultimately matures to a scar during the remodeling phase. It has a thick network of vessels and capillaries, a significant amount of cells—macrophages and fibroblasts as well as collagen fibers of an accidental spatial orientation. It is characterized by a faster metabolism, compared with the dermis, which indicates that the cell in question has an increased energy demand, which, in turn, is connected with cell migration, division, and with an intensified protein biosynthesis [70].

*(b) Epithelialization.* Epithelialization is a multiphase process which is about reconstructing the epithelium after the injury [15]. Epithelial cells, participating in closing the wound surface, originate both from the wound edges and epithelial appendages, such as hair follicles, sweat glands, or sebaceous glands.

The process in question comprises cellular detachment, their migration to the wound area, proliferation, and differentiation [15]. The mediators which stimulate the migration and proliferation of the mentioned cells are the growth factors, such as EGF, KGF, and TGF-*α*. The properties of proliferation are demonstrated only by cells lying directly on a basement membrane [35]. They also “deliver” new cells to the epithelial layer which is being created. The cell migration lasts up to the moment when the epithelial cells are connected and create a uniform layer. TGF-*β* is the only growth factor which accelerates “maturation” of epithelial cell layers [37]. A significant role in the process of keratinocytes separation from their basement membranes is played by the matrix metalloproteinase—MMP-2 (gelatinase-A) and MMP-9 (gelatinase-B) which degrade collagen type IV of the basement membrane and collagen type VII which creates anchoring fibrils. MMP-1 (interstitial collagenase) supports the migration of keratinocytes by a network of collagens types I and III, while stromelysin-1 and stromelysin-2 support the migration of these cells by a network of fibronectin, laminines and glycosaminoglycans.

Epithelialization is a clinical symptom of healing; however, it is not a sign of the end of this process. The final phase of the above-mentioned process is the remodeling of the granulation tissue [15, 69].

*(c) Angiogenesis.* Angiogenesis is a process of creating new blood vessels [7, 34, 75–77]. This process restores blood circulation in the place of damage and prevents the development of ischemic necrosis simultaneously stimulating the tissue repair process. It is stimulated by microenvironment local factors, such as low oxygen tension, low pH, or high lactic acid concentration [38, 78]. Moreover, many soluble mediators, such as bFGF, TGF-*β*, TNF-*α*, VEGF, angiogenin, and angiotropin secreted by epithelial cells, fibroblasts, endothelial cells, and macrophages demonstrate a strong proangiogenic activity [2, 34]. The phenomenon of angiogenesis activation or suppression via hypoxia inducible factor was also described [15].

The regulation of angiogenesis, besides stimulating factors, comprises also factors hindering the process in question. The latter ones are angiostatin and thrombospondin [4, 75]. Proangiogenic activity is exerted by hyaluronic acid molecules of a low molecular mass, while the ones of a big molecular mass exert the opposite activity [79, 80].

Angiogenesis is a key phase of the healing process. In the course of this process, endothelial cells migrate to the temporary matrix of the wound after which, they proliferate and subsequently they create a network branching into a form of tubular structures [13, 35]. The migration of endothelial cells requires a local secretion of matrix metalloproteinases digesting basement membranes and releasing growth factors sequestrated in the ECM [35]. Joining of independent “budding” branches of endothelial cells creates a structure which gives the beginning for a new blood vessel loop. This process lasts until the essential restoration of the capillary system and up to the moment of providing proper oxygen influx and nutrients to the wound environment because of that [35]. Visible capillary tuffs give the wound surface a granular appearance, which is the reason for the expression “granulation tissue.” When the tissue is replaced by collagen matrix and in the last phase by a scar, its “requirements” concerning oxygen influx and nutrients are significantly lower. Angiogenesis is stopped, while a part of capillaries disintegrates during the process of apoptosis. It is a sluggish process and paling of the scar takes many years.

Summing up, the proliferation phase is connected with the activity of fibroblasts which, in the presence of newly formed blood vessels, proliferate and synthesize ECM components. Endothelial cells proliferate and migrate above the granulation tissue “closing” the wound surface.

### 2.4. Remodeling Phase

Remodeling is the last phase of the healing process [35]. In its course, the wound surface is contracted [81]. The key phenomenon of wound contracture is phenotypic differentiation of the preexisting fibroblasts into myofibroblasts [82–84]. The latter ones contain fibrils of alpha smooth muscle actin (*α*-sma) microfilaments, which give the cells the property of contracting [85]. In turn, the integrin receptors *α*_1_ *β*_1_ and *α*_2_ *β*_1_ react with specific places on collagen and mediate in contracting the granulation tissue [82]. The mentioned transformation takes place in the second week of healing, which is why myofibroblasts become the most numerous populations of cells in the granulation tissue [35, 38, 86].

During this phase of the healing process, the granulation tissue “matures” to the form of a scar, which is accompanied by the increase of mechanic strength of the formed tissue. The maturation process of the granulation tissue comprises the reduction of capillary amounts, by aggregating into bigger blood vessels, lowering the content of glycosaminoglycans and proteoglycans as well as the water content connected with glycosaminoglycans and proteoglycans [15, 39]. Cell density and metabolic activity of the tissue are also lowered. The mutual proportion of collagen types changes (type I collagen content increases in favor of collagen type III), the total collagen content increases, its spatial organization becomes arranged, and the number of covalent cross-links increases, which leads to increased tensile strength of the tissue. The tensile strength, in the case of the wound freshly covered with epithelium, equals 25% related to the dermis, while, after many months of reconstruction, the strength equals 80% related to the unchanged tissue [15, 38, 87].

Summing up, during the remodeling phase, the amount of fibroblasts decreases and the vascular density is lowered. The initial scar tissue, characterized by delicate, accidentally organized collagen fibers, typical for proliferation phase, is replaced by a matrix which resembles the dermis in which mature, cross-linked collagen fibers, of the proper diameter, construct a framework of the newly formed tissue [1, 35, 88].

The functions of cells participating in the healing process are regulated by cytokines and growth factors as well as by interactions with ECM components, mediated by integrin receptors and adhesive molecules. Matrix metalloproteinases, which are released by endothelial cells and fibroblasts, enable these cells to migrate, while the neutrophil and macrophage proteases remove degraded matrix components assisting in the remodeling of the initial scar tissue [35].

The fundamental role in the healing process is played by extracellular matrix components. In our previous experimental studies, we proved that ECM constituents, including collagen, glycosaminoglycans, vitronectin, and laminin, turned out to be better effectors of natural therapeutic agent such as propolis than silver sulfadiazine (so-called “gold standard” in topical wound management) in experimental burn wound healing [28, 32, 89]. Estimating the expression of mentioned GAGs during burn treatment with propolis, we observed that the apitherapeutic agent we used accelerates the burned tissue repair by stimulation of the wound bed glycosaminoglycan accumulation needed for granulation, tissue growth, and wound closure. Moreover, propolis accelerates chondroitin/dermatan sulfates structure modification responsible for binding growth factors playing a crucial role in the tissue repair [28, 32]. The role of ECM components in repairing tissue damages is the subject of the in-depth, overview studies [70, 90–93].

In conclusion, ECM components, particularly glycosaminoglycans and proteoglycans, play a fundamental role in wound healing process. Understanding biochemical mechanisms by which ECM components modulate each stage of the process of soft tissue remodeling after injury is of great importance in the description (implementation) of new therapeutic strategies connected with generating a favorable biochemical environment supporting wound healing process.
